# Fasting and Glucose Metabolism Differentially Impact Peripheral Inflammation in Human Type 2 Diabetes

**DOI:** 10.3390/nu16101404

**Published:** 2024-05-07

**Authors:** Gabriella H. Kalantar, Shubh Saraswat, Sara SantaCruz-Calvo, Fatemeh Gholamrezaeinejad, Aida Javidan, Madhur Agrawal, Rui Liu, Philip A. Kern, Xiaohua Douglas Zhang, Barbara S. Nikolajczyk

**Affiliations:** 1 Department of Microbiology, Immunology, and Molecular Genetics, University of Kentucky, Lexington, KY 40536, USA; gabriella.pugh@uky.edu; 2 Department of Biostatistics, University of Kentucky, Lexington, KY 40536, USA; shubh.saraswat@uky.edu (S.S.); douglas.Zhang@uky.edu (X.D.Z.); 3 Department of Pharmacology and Nutritional Sciences, University of Kentucky, Lexington, KY 40536, USAfatemeh.Gholamrezaeinejad@uky.edu (F.G.); aja252@uky.edu (A.J.);; 4 Department of Pharmaceutical Sciences, University of Kentucky, Lexington, KY 40536, USA; 5 Department of Internal Medicine, University of Kentucky, Lexington, KY 40536, USA; pake222@uky.edu; 6 Barnstable Brown Diabetes and Obesity Research Center, University of Kentucky, Lexington, KY 40536, USA

**Keywords:** obesity, systemic inflammation, cytokines, partial least squares discriminant analysis, extracellular flux, Hba1C, glycemic control

## Abstract

Cytokines produced by peripheral T-helper 1/17 cells disproportionately contribute to the inflammation (i.e., metaflammation) that fuels type 2 diabetes (T2D) pathogenesis. Shifts in the nutrient milieu could influence inflammation through changes in T-cell metabolism. We aimed to determine whether changes in glucose utilization alter cytokine profiles in T2D. Peripheral blood mononuclear cells (PBMCs), CD4^+^ T-cells, and CD4^+^CD25^−^ T-effector (T_eff_) cells were isolated from age-matched humans classified by glycemic control and BMI. Cytokines secreted by CD3/CD28-stimulated PBMCs and T_eff_ were measured in supernatants with multiplex cytokine assays and a FLEXMAP-3D. Metabolic activity of stimulated CD4^+^ T-cells was measured by a Seahorse XFe96 analyzer. In this study, we demonstrated that T-cell stimulated PBMCs from non-fasted people with T2D produced higher amounts of cytokines compared to fasting. Although dysglycemia characterizes T2D, cytokine production by PBMCs or CD4^+^ T-cells in T2D was unaltered by hyperglycemic media. Moreover, pharmacological suppression of mitochondrial glucose oxidation did not change T-cell metabolism in T2D, yet enhanced cytokine competency. In conclusion, fasting and glucose metabolism differentially impact peripheral inflammation in human T2D, suggesting that glucose, along with fatty acid metabolites per our previous work, partner to regulate metaflammation. These data expose a major disconnect in the use of glycemic control drugs to target T2D-associated metaflammation.

## 1. Introduction

Loss of glycemic control clinically differentiates obesity from obesity-associated type 2 diabetes (T2D). While glycemic control drugs lower the risk of health complications in people with T2D [1,2,3,4], we have yet to completely define the role of glycemic control in inflammation, a critical mediator of T2D pathogenesis. A number of studies have treated T2D subjects with anti-inflammatory drugs, but saw little improvement in glycemic control, exposing a critical gap in translating the link between systemic inflammation and glycemic control into clinical practice [5,6,7,8]. Immune cells provide essential feedback towards regulating systemic glucose and lipid metabolism by trafficking through insulin-responsive tissues. Some cytokines (e.g., IL-4, IL-13) promote insulin signaling [9,10,11,12,13] and further act to counter the classical proinflammatory cytokines (e.g., IFN-γ, TNF-α, IL-17A/F) that negatively regulate insulin signaling to maintain healthy levels of basal glucose uptake/metabolism [14,15,16,17]. In obesity and T2D, the nutrient milieu is associated with a systemic shift to pro-inflammatory cytokine dominance, further diminishing peripheral insulin responsiveness [18]. This study focuses on cytokine production by CD4^+^ T-cells, based on our demonstrations that T-cells, particularly Th1 and Th17 subsets, disproportionately contribute to peripheral inflammation in T2D [19,20].

T2D is characterized by hyperglycemia, especially when inadequately treated, and some studies report that hyperglycemia promotes aberrant activation and higher responsiveness of peripheral CD4^+^ T-cells [21,22]. Some studies have associated hyperglycemia with higher numbers of peripheral CD4^+^ T-cells, and higher Th1 and Th17 subset frequencies [23,24,25,26]. On the other hand, our lab and others, in part through comprehensive flow cytometry panels, have demonstrated that CD4^+^ T-cells and effector CD4^+^ T-cells (T_eff_) subsets are similarly frequent in peripheral blood from T2D compared to obesity plus normal glucose tolerance (ObNGT) subjects, despite higher production of Th1 and/or Th17 signature cytokines [18,20,27]. These data highlight the importance of cellular function over frequency in potentiating meta-inflammation in T2D.

Some circulating and tissue cytokines increase post-prandially, highlighting the direct relationship between changes in exogenous nutrient levels and systemic cytokine production in non-T2D people [28]. Post-prandial increases in serum IL-6, a clinical marker of the inflammation that predicts T2D risk/severity, are reported consistently, but other studies have noted increases in TNF-α, IL-1β, and C-reactive protein after a meal [28,29]. None of these studies included people with T2D, whose glycemic control varies more radically than those without T2D. Furthermore, these studies did not test cytokine production by peripheral immune cells, despite numerous limitations of using serum cytokines to define inflammation [30,31,32]. Solely focusing on serum cytokines may be less clinically useful than originally posited, given that targeting individual cytokines associated with T2D pathogenesis, like IL-1β and TNF-α, did not improve insulin sensitivity or T2D onset in clinical trials [5,33,34].

New data show that ex vivo cytokine production by T-cell-stimulated PBMCs from subjects with T2D is higher in cells from fed compared to fasted individuals. We further show that in vitro hyperglycemic treatment of cells from fasted subjects does not recapitulate T2D-associated changes in cytokines characteristic of immune cells from fed compared to fasted T2D subjects. Peripheral CD4^+^ T-cells from T2D subjects were metabolically more active, as demonstrated by higher oxidative metabolism compared to cells from ObNGT and lean NGT subjects. However, blocking mitochondrial pyruvate import and thus the pathways necessary for mitochondrial glucose metabolism did not alter mitochondrial oxidative phosphorylation (OXPHOS) of cells from lean NGT or T2D subjects. This result indicates a role for pyruvate oxidation in metabolism of cells from ObNGT subjects. In contrast to the demonstration that fasting lowers cytokine production compared to fed conditions, cytokine production from T_eff_ cells was enhanced by preventing mitochondrial pyruvate oxidation. Our data are consistent with the preferential use of pyruvate for the T2D-associated metabolism that fuels the inflammation characteristic of CD4^+^ T-cells from people with T2D, and fundamentally differs from fasting-induced changes in cell function. Our data raise the possibility that dysglycemia partners with changes in glucose metabolism and thus a different array of metabolites to reinforce the previous demonstration of pro-inflammatory changes in fatty acid flux in T2D [35].

## 2. Materials and Methods

### 2.1. Human Subjects

Subjects were divided into three groups, defined as (1) lean (BMI 18–25) with normal glucose tolerance (NGT); (2) obese (BMI 27–40; ObNGT); and (3) obese with T2D (BMI 27–40). For this cohort, T2D was indicated by HbA1C, fasting blood glucose, and/or 2-h oral glucose tolerance, as categorized by the American Diabetes Association (Table S1). Some subjects with T2D had values in the normal range of glycemic control because they were effectively treated with glycemic control medications; these are not shown to avoid confusion. Glycemic control prior to T2D treatment was generally measured outside the University of Kentucky Health care system and thus the data were unavailable. Inclusion and exclusion criteria were previously published [20]. Medications are listed in Table S2. Fasting was defined as no calorie intake for ≥12 h and was self-reported. Non-fasting subjects by this criteria were termed “fed”, although the time of the last meal was not indicated. Most, although not all, blood draws occurred in the morning and individual chronotype was not determined. Thus, circadian effects were not investigated.

### 2.2. Cell Manipulation and Cytokine Measurements

PBMCs and T_eff_ cells (CD4^+^CD25^−^) were isolated, archived, and thawed, as previously published [20,36]. Total CD4^+^ T-cells were isolated from PBMCs using a negative selection kit for human CD4^+^ T-cell and an LS column in a MidiMACs Separator (Miltenyi, Bergisch Gladbach, Germany). For glucose titration studies, PBMCs and total CD4^+^ T-cells were resuspended at 10^6^ cells/mL in RPMI-1640 medium containing 4 mM L-glutamine and 25 mM HEPES (Gibco, Grand Island, NY, USA), and supplemented with 10% heat-inactivated FBS, 1% penicillin–streptomycin (Gibco), and 1% Na-pyruvate (Corning, Corning, NY, USA) (termed ‘complete RPMI’, herein). Complete RPMI was either glucose-free and supplemented with 5 or 11 mM of the osmolarity control 3-O-D-methyl-D-glucopyranose (3OMDG; Sigma Aldrich, St. Louis, MA, USA) or 5 mM or 11 mM D-glucose (Gibco) to replicate physiological in vivo euglycemia (5 mM/L = 90 mg/dL) or hyperglycemia (11 mM/L = 200 mg/dL), respectively. For metabolic flux analysis and secreted cytokine measurements under pharmacological suppression of the mitochondrial pyruvate carrier (MPC) with UK5099 (Tocris, Bristol, UK), total CD4^+^ T-cells and T_eff_ cells were resuspended in complete RPMI containing 11 mM D-glucose. PBMCs, total CD4^+^ T-cells, and T_eff_ cells were cultured for 40 h in the presence or absence of CD3/CD28 Dynabeads (Gibco) at 1 bead/cell as indicated. Consistent with numerous prior studies, total CD4^+^ T-cells and T_effs_ produce few to no cytokines in the absence of stimuli [27,36]. Aliquots of supernatants were frozen and thawed 1–2 times for 25-plex cytokine measurements using a Th17 kit (Millipore Sigma, Burlington, MA, USA). Cytokine signal was read using a FlexMAP 3D with Luminex xPONENT and Bio-plex Manager software v 6.1 (Bio-Rad, Hercules, CA, USA), as previously published [20].

### 2.3. Metabolic Flux Analysis

Total CD4^+^ T-cells were cultured in complete RPMI containing 11 mM D-glucose for 40 h in the presence of CD3/CD28 Dynabeads and either vehicle (DMSO, Sigma, San Diego, CA, USA) or 2 μM UK5099. Aliquots of supernatants were stored for bioplex analysis, as described above. Cells were resuspended in Seahorse XF DMEM containing 10 mM glucose, 4 mM L-glutamine, and 1% Na-pyruvate (Agilent, Santa Clara, CA, USA) at a density of 250 K cells/175 μL and plated in triplicate or quadruplicate/condition in 96-well cell culture microplates (Agilent) coated with poly-D-lysine (Sigma Aldrich). Plated cells were incubated for 1 h at 37 °C without CO_2_. Oxygen consumption rate (OCR) and extracellular acidification rate (ECAR) were measured by the Seahorse XFe96 analyzer (Agilent) using Wave software v 2.6.3.5 and a mitochondrial stress test that includes injections of oligomycin (Calbiochem, San Diego, CA, USA), FCCP (Enzo, Lausanne, Switzerland), and a combination of rotenone and Antimycin A (Enzo) at final concentrations of 3 μM, 1 μM, and 14 μM (each), respectively, as previously published [27,35].

### 2.4. Statistical Analysis

We used GraphPad Prism for one-way or two-way ANOVA with Bonferroni’s multiple comparisons and a 95% confidence interval to assess cohort- or treatment-specific differences in cytokine competency, and mitochondrial stress test parameters. Paired *t*-tests were used to assess cohort- or treatment-specific differences in OCR and ECAR. A Mann–Whitney U test was used to assess differences in cytokine competency between fasted and fed conditions. *p* < 0.05 indicated significant differences.

### 2.5. Bioinformatics

For the partial least square discriminant analysis (PLSDA) [20,37,38], cytokine data were log-transformed to mitigate skewing. Cytokines with a small range of values had the option to be as (or more) important as cytokines with a large range of values. We thus scaled each cytokine to zero mean and unit variance before applying PLSDA. We excluded 7 cytokines (IL-15, -1b, -33, -17E, -25, -27, -31, and -28A) from analyses because their concentrations were lower than the minimal detectable value in every sample. Cytokine production by resting cells was below the level of detection in ≥50% of the samples (not shown) and thus was not informative. After all data preprocessing (based on exploratory analysis), PLSDA was applied mainly using the R function splsda() in the mixOmics package [39] and R version R4.2.2. We used the first two PLSDA components (linear combination of cytokines derived from the PLSDA analysis) for each comparison as indicated to display the classification results based on the cytokines with variable importance projection (VIP) scores greater than 1. The PLSDA components are ordered from the largest at the bottom to the smallest at the top, based on their contributions to the covariances between the responses and the cytokines.

## 3. Results

### 3.1. Fasting Changes Immune Cell Cytokine Profiles in T2D

**Figure 1:**
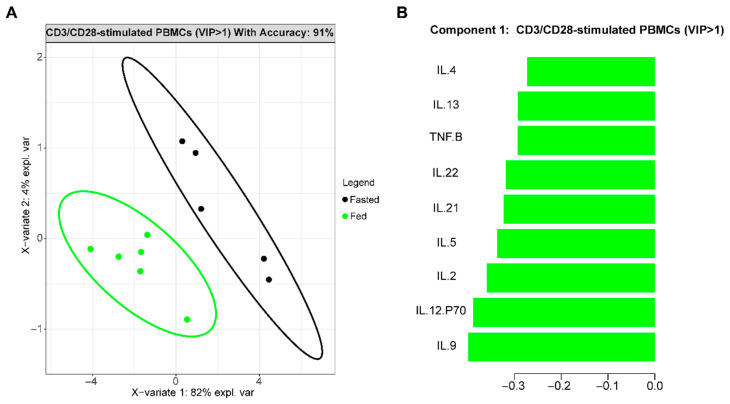
T-cell-stimulated (CD3/CD28) PBMCs from fasted and fed subjects with T2D produce different cytokine profiles. (**A**): PLSDA 2D projection of fasted (*n* = 5) and fed (*n* = 6) T2D subjects based on cytokine production generated a highly accurate model (91%). (**B**): Top ranked cytokines from PLSDA with a VIP Score > 1 that distinguished fasted and fed subjects’ cells.

Fasting changes the abundance of numerous macromolecules in circulation, in part by shifting many organs/cell types from glucose towards lipid metabolism in metabolically flexible individuals. Obesity lowers metabolic flexibility, with further metabolic decline leading to the chronic hyperglycemia that defines T2D [40,41,42,43]. To quantitate the impact of fasting on immune cell cytokine production, we purified PBMCs from people with T2D at two time points: (1) fasted overnight, or (2) not fasted (i.e., “fed”), and measured cytokine production following T-cell targeted stimulation. We used PLSDA to indicate overall “inflammation”, and thus separate samples from fasted and fed subjects (Figure 1A). Ranking of cytokines from most to least important for differentiating fasting and fed inflammation identified 12 cytokines (VIP) with a score ≥ 1 produced in higher amounts by cells from fed compared to fasted subjects (Figure 1B, green; Figure S1). All differentiating cytokines were characteristic of non-Tregs, (herein referred to as T_eff_). Together, these data support previous demonstrations of post-prandial increases in immune cell cytokine production [29], and highlight that cytokine competence, or the ability of T-cell-stimulated PBMCs to make cytokines, is lower under fasting conditions in T2D. Fasting-induced shifts in cytokine profiles generated by PBMCs from normoglycemic subjects with obesity (ObNGT) predicted fasting/fed status less reliably (Figure S1D, 70%) than models generated by T2D subjects’ cells (Figure 1A, 91%). Specific cytokines that were disproportionately important for defining the fed state in ObNGT (Figure S1E: CCL20-20, IL-17F, IFNγ, etc.) differed from those most important for defining the fed state in T2D (Figure 1B). These data raise the possibility that key differences in T2D and ObNGT subjects, including dysglycemia, impact the shift of inflammatory responses in the fasting compared to the fed state.

### 3.2. In Vitro Glucose Concentrations Do Not Alter Cytokine Production by Cells from Fasted Donors

**Figure 2:**
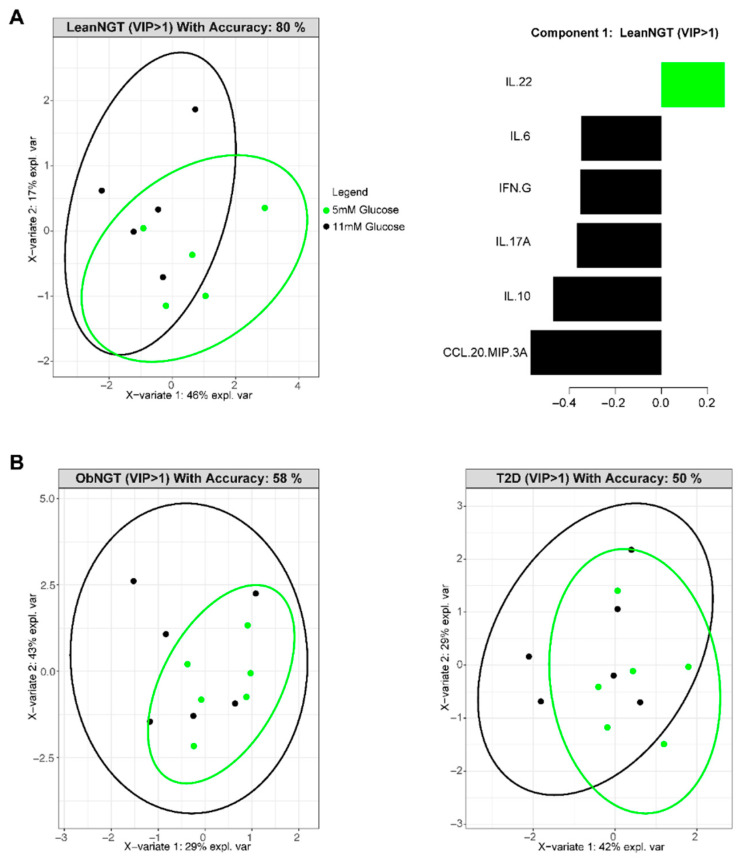
Hyperglycemia in vitro does not alter cytokine production in CD3/CD28-stimulated PBMCs from fasted subjects with obesity/T2D. (**A**,**B**): PLSDA 2D projection of cytokine production from PBMCs of a cohort with a lean BMI (*n* = 5) or obesity BMI (*n* = 6) with normal glucose tolerance/NGT or a cohort with obesity and T2D (*n* = 6). Cells were activated with CD3/CD28 beads for 40 h in the presence of either 5 mM (green dots) or 11 mM (black dots) D-glucose. An accuracy of ≥70% indicates a predictive model. (**A**) (right): PLSDA-ranked cytokines in the most-distinguishing component (component 1) for those produced by cells from the leanNGT cohort with a VIP score ≥ 1. Ranking is from most predictive (bottom) to least predictive (top) of 5 mM (black) or 11 mM (green) glucose conditions.

To test how glucose excursions characteristic of metabolic decline affect immune cell function, as measured by cytokine profiles, we stimulated PBMCs from all three cohorts (LeanNGT, ObNGT, and T2D) in vitro in the presence of physiologically high and low glucose (or osmolarity controls) and quantitated cytokines. PLSDA 2D projections of cytokine production by PBMCs from leanNGT subjects showed that glucose impacted cytokine profiles, as evidenced by higher production of multiple cytokines in 11 mM (modeling T2D hyperglycemia) compared to 5 mM (normoglycemia) glucose (Figure 2A, left). Hyperglycemia-induced cytokines included T1/Th17 cytokines (IFN-γ, IL-17A, and CCL-20) that echo a T2D profile [19] (Figure 2A, black bars, right). In contrast, hyperglycemia did not impact cytokine production by cells from ObNGT and T2D subjects (Figure 2B), and nor did the osmolarity control 3OMDG (Figure S2A). Models with both glucose amounts plus both osmolarity controls, and univariate analysis of cytokine production by total CD4^+^ T-cells from ObNGT subjects, were not informative (Figure S2B). We conclude that physiologically high glucose promotes Th1/Th17 cytokine production only in the absence of obesity/T2D.

### 3.3. Mitochondrial Glucose Oxidation Regulates Cytokine Production while Modestly Raising OXPHOS in T-Cells from Donors with T2D

**Figure 3:**
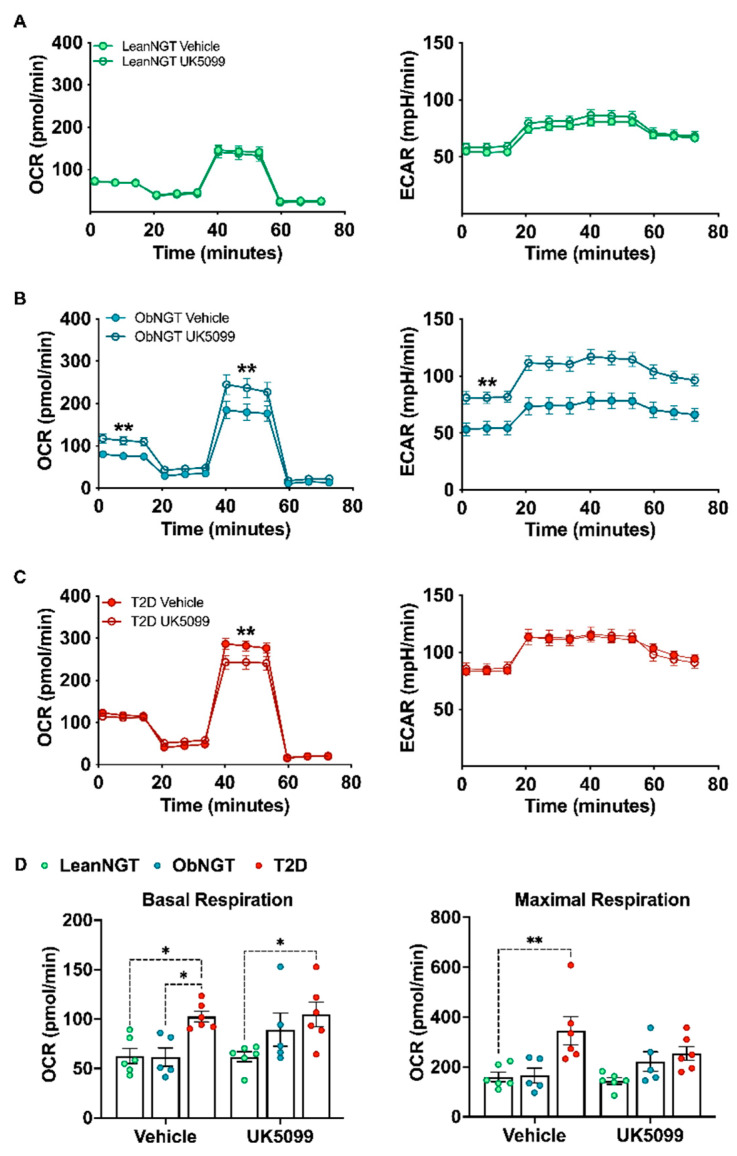
Inhibition of mitochondrial pyruvate import inversely changes oxidative metabolism in CD4^+^ T-cells, depending on glycemic control but not BMI. Oxygen consumption rate (OCR) and extracellular acidification rate (ECAR) of total CD4^+^ T-cells from a cohort with (**A**) lean BMI (*n* = 6, green dots) or (**B**) obese BMI (*n* = 5, blue dots) with NGT or (**C**) obesity and T2D (*n* = 6, red dots) that were activated with CD3/CD28 beads for 40 hr in the presence of either vehicle or UK5099. (**D**) Basal and maximal respiration with vehicle or UK5099 treatment. Differences in basal and maximal OCR between treatments (**A**–**C**) were assessed by a paired *t*-test using the group means at measurements 0–20 min (basal) and 40–60 min (maximal). Differences in ECAR between treatments within each cohort were assessed by a paired *t*-test of the average ECAR of measurements 1–3 (0–18 min). Differences in panel D were assessed using sample means by a two-way ANOVA and Bonferroni’s multiple comparisons. *: *p* < 0.05, **: *p* < 0.01.

CD4^+^ T-cells are the major source of cytokines potentiated by feeding in T2D (Figure 1), and high glucose treatment of leanNGT cells increases production of multiple CD4^+^ T-cell cytokines (Figure 2A). We therefore tested the role of glucose metabolism in mitochondrial OXPHOS and cytokine production by blocking mitochondrial oxidation of pyruvate, a downstream metabolite of glucose. We used UK5099 to block mitochondrial pyruvate import and thus encourage glucose-derived pyruvate to be reduced to lactate rather than oxidized through the TCA cycle. UK5099 had no impact on oxygen consumption rate (OCR), a measure of mitochondrial oxidative phosphorylation (OXPHOS), in T-cells from metabolically flexible LeanNGT subjects (Figure 3A). In contrast, UK5099 increased OCR in T-cells from ObNGT subjects (Figure 3B) to approximate the overall higher OCR of T2D cells (Figure 3C and Figure S3A). Extracellular acidification rate (ECAR), an indicator of pyruvate reduction, was higher in activated T-cells from T2D subjects (Figure S3B), indicating that high metabolism distinguished cells from T2D and NGT subjects irrespective of BMI of NGTs. Differently from results from ObNGT cells, UK5099 decreased maximal OCR in T-cells from subjects with T2D (Figure 3C), highlighting roles for pyruvate oxidation in maintaining T-cell OXPHOS. These data indicate distinct pyruvate contributions to CD4^+^ T-cell OXPHOS in obesity +/− T2D, although this effect of UK5099 was diluted by multiple comparisons (Figure 3D). OCR generated by T-cells stimulated in the presence of UK5099 remained higher for the T2D group compared to the lean NGT group (Figure S3C), suggesting that non-glucose fuel sources maintain high OXPHOS in T2D T-cells. As shown in Figure 3B, OCR increases for ObNGT cells treated with UK5099 to comparable levels of the T2D group with the same treatment (Figure S3C), thus indicating that a switch to non-glucose fuels in the presence of obesity could be a key event in the metabolic reprogramming of T-cells in T2D pathogenesis. Surprisingly, UK5099 increased ECAR only in ObNGT T-cells (Figure 3B, right panel), suggesting metabolic flexibility was higher in both leanNGT and T2D cells. These data show that favoring reductive over oxidative pyruvate metabolism with UK5099 alters glucose utilization and OXPHOS in CD4^+^ T-cells, and that outcomes are modified by metabolic status of the cell donor. This Interpretation is consistent with the possibility that glycemic control drugs in T2D improve fuel flexibility, and that glucose or other mitochondrial fuels generate cohort-modified metabolites that are not detected by Seahorse.

**Figure 4:**
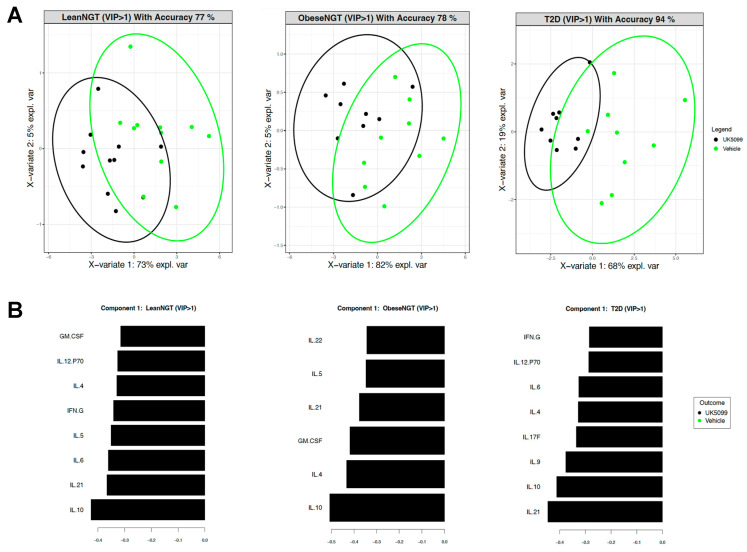
Inhibition of mitochondrial pyruvate import enhances cytokine production from T_eff_ cells of all cohorts. (**A**) PLSDA 2D projections of cytokine production from T_eff_ cells stimulated in the presence of vehicle (green dots) or UK5099 (black dots) from LeanNGT (*n* = 11, accuracy: 77%), ObNGT (*n* = 9, accuracy: 78%), and T2D subjects (*n* = 9, accuracy: 94%). (**B**) PLSDA-ranked cytokines in the most-distinguishing component (component 1) per cohort with a VIP score ≥ 1 ranked from most predictive (bottom) to least predictive (top) for vehicle or UK5099. Black bars indicate cytokines were produced at higher effective concentrations in the UK5099 cultures.

To test the impact of blocking pyruvate oxidation without changing OXPHOS, we profiled cytokines produced by the more inflammatory CD4^+^ T-cell subset, T_eff_, following stimulation in the presence/absence of UK5099. Figure 4 shows that UK5099 broadly increases cytokine production by T_eff_ from all three subject cohorts, with extensive overlap in cytokines dominating the response to UK5099 amongst the cohorts. Most notable are IL-10 and IL-21, two pleiotropic cytokines with classical “pro” and “anti”-inflammatory properties. Comparison of cytokine production by T2D immune cells using different methods of changing fuel utilization (Figure 1B and Figure 4), interpreted in the context of outcomes from previous glucose deprivation studies [27], indicate that blocking glucose oxidation activates, while fasting lowers, production of an overlapping set of T-cell cytokines. These data indicate that glycolysis does not recapitulate the anti-/non-inflammatory effect of fasting, and that the use of glycemic control as a surrogate for metabolic health sub-optimally estimates risk for inflammatory comorbidities of obesity, including T2D.

## 4. Discussion

Glucose is an essential fuel source for immune cell proliferation and survival [44,45,46,47]. Hyperglycemia in T2D is hypothesized to cause increased peripheral CD4^+^ T-cell flux, aberrant activation and effector functions, and impaired migration [24,25,26]. Additionally, the metabolic reprogramming paradigm states that T_eff_ differentiation and function rely on preferential utilization of glycolysis, defined as pyruvate conversion to lactate in the presence of oxygen [48,49,50]. We tested the hypothesis that the hyperglycemia characteristic of T2D drives T-cell inflammation as measured by cytokine production. Our data contradict this hypothesis by showing that (1) hyperglycemia alone has no impact on ex vivo cytokine production in T2D (Figure 2); (2) forcing glycolysis (with UK5099, as defined above) lowers T-cell mitochondrial metabolism specifically in T2D (Figure 3), but raises cytokine production by cells from all cohorts (Figure 4). Thus, the only T2D-specific role for glucose in T-cells we found was to support mitochondrial metabolism. The effect of mitochondrial pyruvate carrier (MPC) inhibition by UK5099 on total CD4^+^ T-cells and T_eff_ cells suggested that the metabolic reprogramming paradigm to describe mechanisms fueling T_eff_ function is oversimplified, and that shifts in fuel utilization, perhaps coupled with changes in metabolite generation, play critical roles in the glucose-associated mechanisms that control T-cells. Our data further indicate that changes in glucose oxidation do not recapitulate the complex physiological changes imparted by fasting in subjects with T2D.

Higher concentrations of CD4^+^ T-cell-associated cytokines are produced by PBMCs from fed compared to fasted subjects with T2D, supporting previously published data on post-prandial increases in cytokine production [28,29], but with novelty added by the focus on T2D in our work. The demonstration that physiologically high glucose does not recapitulate the effects of feeding on cytokine production by cells from fasting subjects with T2D (comparing Figure 1 and Figure 2B), indicates that glucose-independent mechanisms, which may include functional fatty acid metabolites, are required for feeding-induced changes in T-cell cytokine profiles in T2D. We previously demonstrated that T2D PBMCs use pyruvate reduction over fatty acid oxidation for cellular metabolism, and that products of fatty acid metabolism support Th17/Th1 function in T2D [27]. The current study expands this model by showing that pyruvate oxidation changes cytokine profiles despite little to no change in metabolism, as measured by OCR and ECAR. These data suggest that glucose metabolites may complement fatty acid metabolites to support T-cell inflammation in T2D. This complementarity between glucose and fatty acid metabolites is further indicated by the ability of high glucose alone to generate a partial Th1/Th17 (T2D-like) T-cell profile in cells from leanNGT subjects. The inability of hyperglycemia to change cytokine profiles in ObNGT or T2D cells from fasting subjects indicates that the impact of glucose is modified by BMI-dependent changes in subjects’ physiology. Finally, the partial overlap but opposing direction of changes in cytokine profiles generated by (1) PBMCs from fasted vs. fed T2D subjects; and (2) T2D T-cells unable to oxidize glucose (e.g., mimicking a glucose fast from a mitochondrial perspective), e.g., changes in IL-21, -9, -4, -12, are consistent with the interpretation that both glucose and fatty acid-derived metabolites are required for T-cell inflammation in T2D. Together, multiple lines of evidence support the model wherein glucose and fatty acid metabolites partner to drive T2D-associated inflammation. Given that ObNGT recapitulates only some of the characteristics of T2D, we speculate that glucose initially promotes metabolic changes in T-cells from people with obesity, with inflammatory changes becoming more pronounced with further loss of glycemic control.

The lack of effect of MPC inhibition on metabolism in CD4^+^ T-cells from the T2D cohort indicates that these cells always prefer pyruvate reduction to generate ATP. This interpretation is consistent with our demonstration that CD3/CD28 (e.g., T-cell)-stimulated PBMCs from T2D compared to ObNGT subjects had higher ECAR but similar OCR. Etomoxir, an inhibitor of CPT1a and thus of long chain fatty acid oxidation, increased ECAR in CD3/CD28-stimulated PBMCs from ObNGT but not T2D subjects [27], which is also consistent with the preference of T2D T-cells for non-mitochondrial metabolism in our current study. Additionally, glycemic control drugs like metformin have been shown to lower the activity of lactate dehydrogenase (LDH) [51], which catalyzes the reduction of pyruvate to lactate during glycolysis. Metformin usage in T2D subjects could account for lack of changes in ECAR with MPC inhibition, suggesting that glycemic control drugs may improve glucose flexibility in T-cells; however, restriction of LDH activity by metformin has been demonstrated in cancer cells, and has yet to be confirmed in T-cells. Given the limitations of Seahorse for identifying significant shifts in TCA cycle fuels amidst similar OCR measures, stable isotope tracer studies will be key for pinpointing the differences in glucose and fatty acid metabolites suggested by our cumulative results, and design of studies testing how metabolites regulate T-cell function in obesity and T2D.

## 5. Limitations and Conclusions

A lack of diverse racial groups and sex considerations (the latter a societal challenge for obesity studies that disproportionately interest women) impact translatability of the work, and the disproportionate prevalence of T2D in males compared to females is also not represented [52]. Na-pyruvate was supplemented to low levels in the cell culture medium to prevent extreme cell death and this could account for the lack of changes in the absence of glucose for some approaches. An additional limitation is that the majority of subjects with T2D are being treated with metformin, which may impact ex vivo T-cell biology, as we have previously published [53]. Metformin at physiological concentrations increases mitochondrial respiration of hepatocytes from people with obesity [54], but lowers high OXPHOS of CD4^+^ T-cells from older lean subjects [53], with analysis of CD4^+^ T-cells from ObNGT subjects in the ongoing ANTHEM metformin trial [55]. Demonstrations of high OXPHOS in CD4^+^ T_eff_ cells in metformin-naïve people with prediabetes [36] suggest that high T-cell OXPHOS may be a characteristic feature of T2D immunopathogenesis in the absence of metformin. Ethical considerations prohibit the stoppage of the T2D drugs, or treatment of healthy people with these drugs for research purposes; in vitro treatments are highly unlikely to mirror impacts of these drug combinations on the immune cells. Thus medication impacts, especially prominent in the T2D cohort, cannot be ascertained. Limitations in sample size for statistical approaches were complemented by bioinformatic modeling, although the importance of predictions from these models will be further established by future follow-ups.

Overall, our data support the conclusion that the effect of fasting on the chronic T cell inflammation of obesity, alone or in the presence of T2D, is not recapitulated by changes in glucose metabolism. This conclusion is consistent with previous demonstrations that changes in fatty acid flux, rather than glucose metabolism, impact T2D-associated systemic inflammation [17]. From a practical perspective, we conclude that the fasting state of subjects who provide blood for research assessments of inflammation must be consistent to support strong interpretations. Finally, we conclude that media glucose has little impact on measures of inflammation, alleviating some concerns about the artificial nature of cell culture as a major determinant of outcomes from meta-flammation studies. This combination of technical and biological insights informs interpretation of similarly translational studies towards more effective design and biomarker selection for clinical trial success.

## Data Availability

The raw data supporting the conclusions of this article will be made available by the authors on request.

## Supplementary Materials

The following are available online at https://www.mdpi.com/article/10.3390/nu16101404/s1. Table S1: Description of human donors, Table S2: List of medications and smoker status; Figure S1: Cytokine production by PBMCs from fed compared to fasting T2D or ObNGT subjects; Figure S2: Physiological glucose concentrations and osmolarity controls in vitro do not alter cytokine production from activated PBMCs or total CD4^+^ T-cells in obesity/T2D. Figure S3: Blocking mitochondrial pyruvate import enhances cytokine production from Teff cells, irrespective of glycemic control or changes to metabolism in total CD4^+^ T-cells.
