# Supplementary material for: Fasting and Glucose Metabolism Differentially Impact Peripheral Inflammation in Human Type 2 Diabetes
Source: Nutrients. 2024 May 7;16(10):1404. doi: 10.3390/nu16101404 (PMC11124302; doi:10.3390/nu16101404)
Supplement: Supplementary file 1 [file nutrients-16-01404-s001.zip › nutrients-2939256-supplementary.pdf]

SUPPLEMENTARY MATERIALS

Table S1. Description of human donors

	Lean BMI + NGT	Overweight/obesity + NGT	Overweight/obesity + T2D
Age, years [median(range)]	57 (36 – 67)	49 (36 – 63)	52 (37 – 64)
BMI, kg m^2 [median(range)]	22.9 (18 – 24)†	31.3 (27 – 43)	35.3 (26 – 40)
FBG, mg dl^- [median(range)]	98 (93 – 98)<br>n = 3	93.5 (88 – 98)<br>n = 13	*N/A<br>*n = 0
A1c, % [median(range)]	5.4 (5.2 – 5.4)<br>n = 3	5.4 (4.7 – 5.5)<br>n = 13	7 (5.5 – 10.8)‡<br>n = 25
Total *N*	17	13	26
Females	13	9	13
Males	4	4	13
White	16	12	18
Black/AA	0	1	8
Native American	1	0	0

*FBG is not measured for subjects with T2D. Clinical diagnosis of T2D was sufficient for subjects on glycemic control drugs; NGT was confirmed in some subjects based on 2hr oral glucose tolerance test blood glucose measures (not shown).

†Indicates a significant difference between LeanNGT and ObNGT or T2D subjects and ‡significant difference between T2D and LeanNGT or ObNGT subjects based on Student's t-test ($P < 0.05$)

Table S2. List of medications and smoker status

Medications	LeanNGT	ObNGT	T2D
Metformin (*N)*	0/17	0/13	19/26
Thiazolidinediones (*N*)	0/17	0/13	1/26
DPP-4 Inhibitors (*N*)	0/17	0/13	2/26
Sulfonylureas (*N)*	0/17	0/13	5/26
Statins (*N*)	1/17	1/13	17/26
ACE Inhibitors (*N)*	0/17	1/13	7/26
Smoker (*N*)	0/17	0/13	0/26

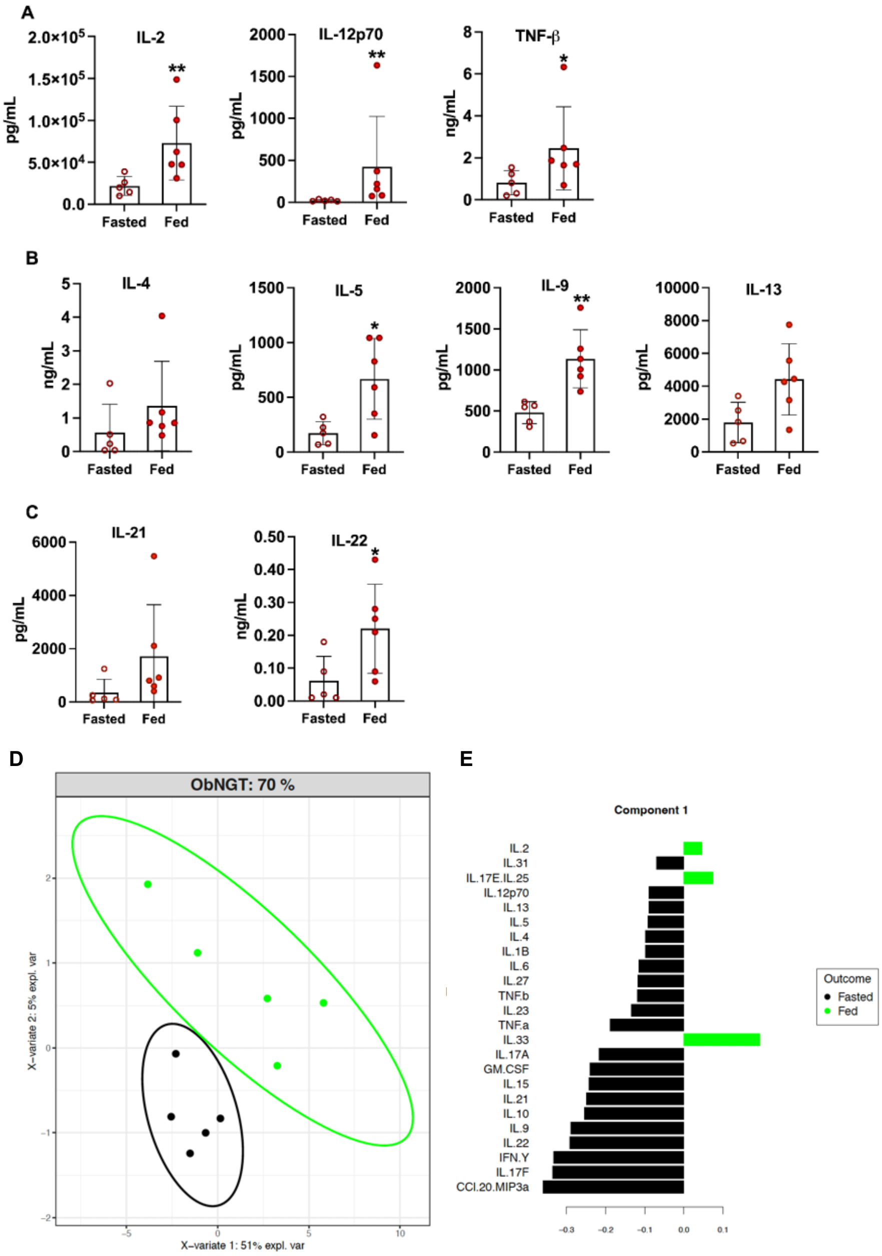

Figure S1. Cytokine production is higher by PBMCs from fed compared to fasting T2D and ObNGT subjects. (A-C) Cytokines from Fig 1B in CD3/CD28-stimulated cells from fasted (n = 5, open circles) and fed (n = 6, closed circles) subjects with T2D. Cytokines for stimulated cells are categorized by Th1 **(A)**, Th2 **(B)**, and Th17 **(C)** according to the literature. Differences were assessed by a Mann-Whitney U test. *: $p < 0.05$, **: $p < 0.01$. **(D)** Cytokine production by T cell-stimulated (CD3/CD28) PBMCs from normoglycemic subjects with obesity (ObNGT) who were fasted (black dots) or fed (green dots) as analyzed by partial least squares analysis (PLSDA). The same N=5 subjects provided samples on two different days: (1) after a >12 hr fast or (2) after a morning meal. All samples were collected at 8-10a. **(E)** Top-ranked cytokines from PLSDA with a VIP Score >1 that distinguished Fed and fasted subjects' cells. The four cytokines that characterize a fed state in T2D subjects (Figure 1; IL-9, IL-12p70, IL-2, and IL-5) did not dominate a fed state in ObNGT.

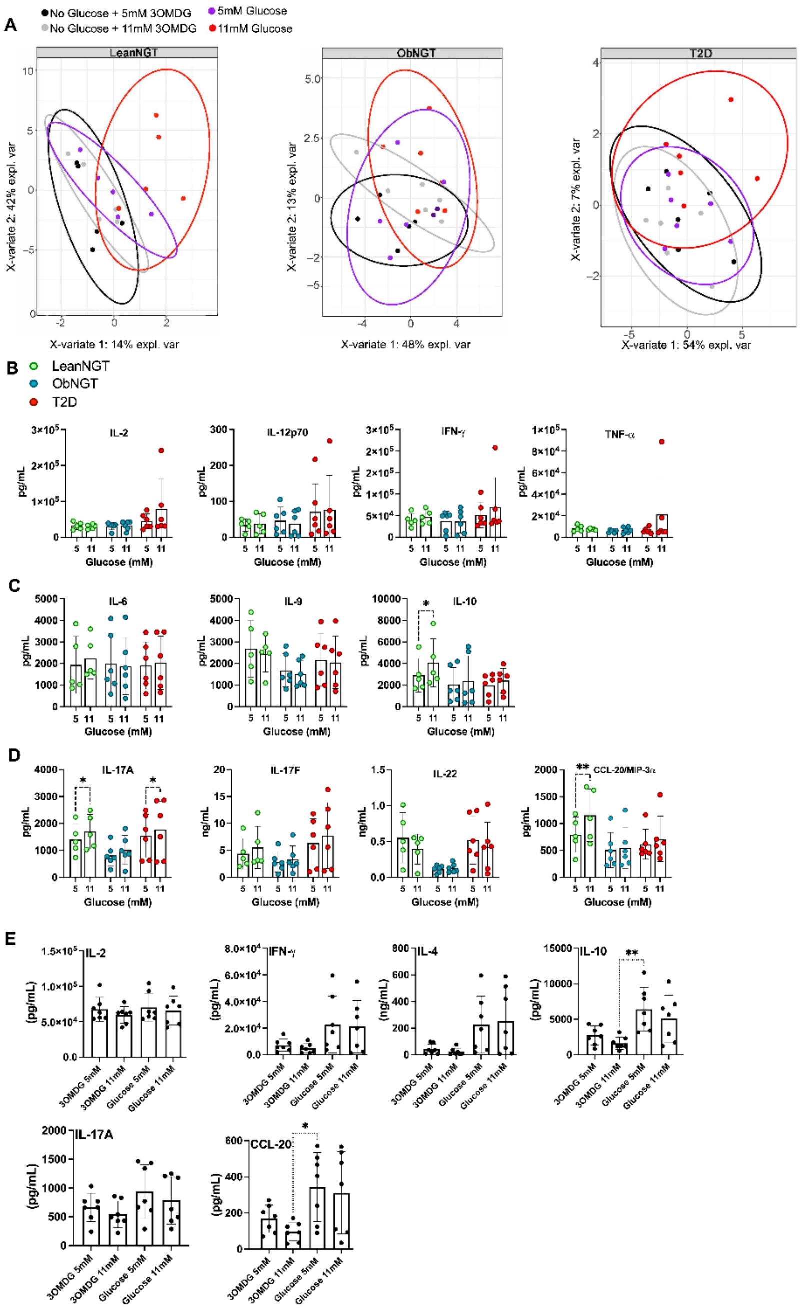

Figure S2. Physiological glucose concentrations and osmolarity controls *in vitro* do not alter cytokine production from activated PBMCs or total CD4⁺ T cells in obesity/T2D.

(A) PLSDA 2D projections of cytokine production from T-cell stimulated PBMCs treated with no glucose + 5mM or 11mM osmolarity control 3-O-Methyl-D-glucopyranose (3OMDG) (black and gray dots, respectively), 5mM glucose (purple dots), or 11mM glucose (red dots). **(B-D)** Representative cytokines of Th1 **(B)**, Th2 **(C)**, and Th17 **(D)** cells under 5 and 11mM glucose conditions. LeanNGT (n = 5, green dots), ObNGT (n = 6, blue dots), and T2D (n = 6, red dots). **(E)** Total CD4⁺ T cells, from subjects with obesity and NGT (n = 7), were stimulated in media containing either no glucose and 5mM or 11mM 3OMDG, 5mM glucose, or 11mM glucose. Differences were assessed by a two-way ANOVA (**B-D**) or one-way ANOVA **(E)** and Bonferroni's multiple comparisons. *: $p < 0.05$, **: $p < 0.01$

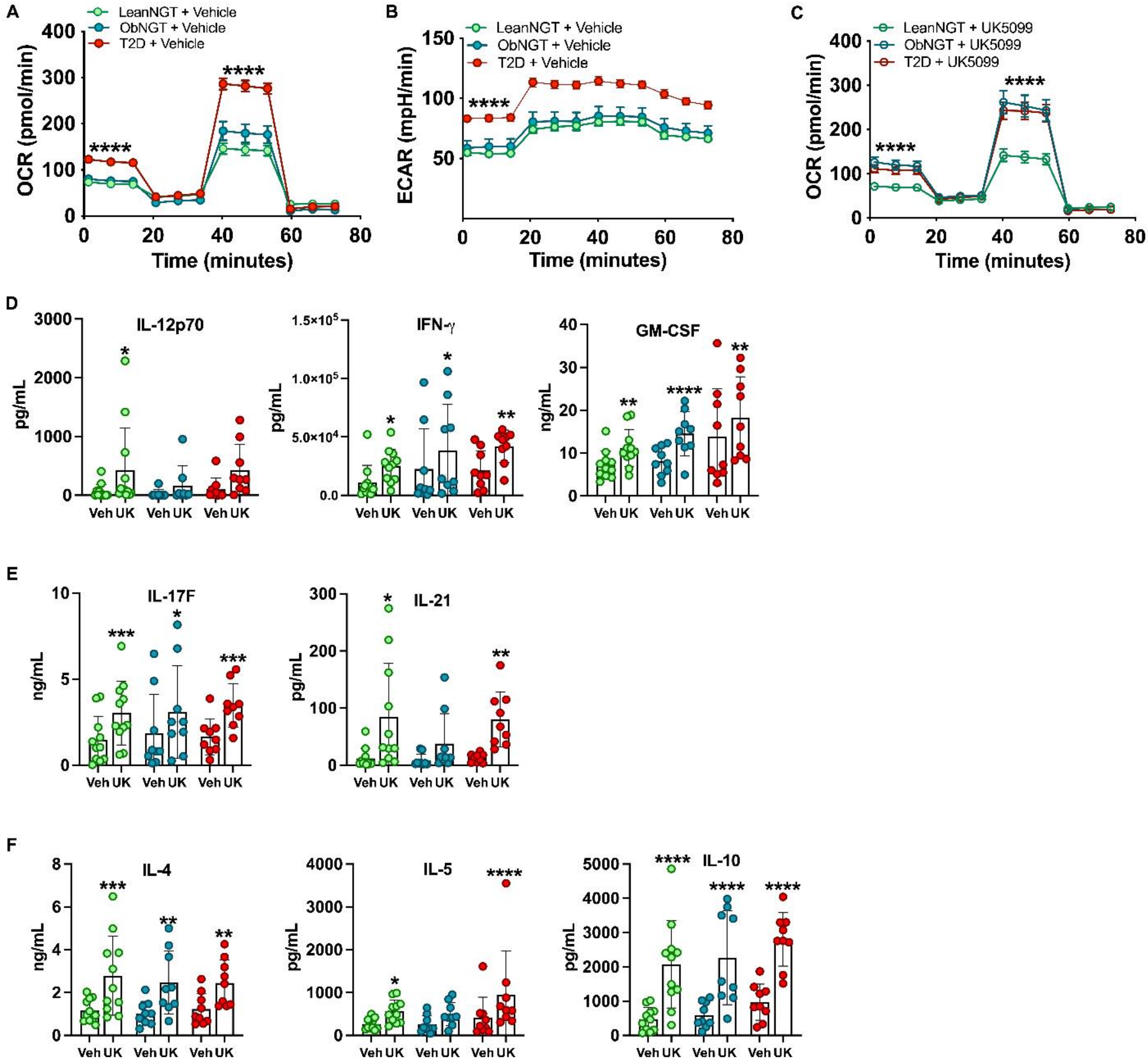

Figure S3. Blocking mitochondrial pyruvate import enhances cytokine production from Teff cells, irrespective of glycemic control or changes to metabolism in total CD4⁺ T cells.

(A) OCR plot of a mitochondrial stress test comparing total $CD4^+$ T cells from LeanNGT (n = 6), ObNGT (n = 5), and T2D (n = 6) donors treated with vehicle (filled green, blue, and red dots, respectively). **(B)** ECAR plot of comparison of cells from LeanNGT (green dots), ObNGT (blue dots), and T2D (red dots) donors treated with vehicle. **(C)** OCR plot of a mitochondrial stress test comparing cells from LeanNGT, ObNGT, and T2D donors treated with UK5099 (open green, blue, and red dots, respectively). **(D-F)** Representative cytokines of Th1 **(D)**, Th17 **(E)**, and Th2 **(F)** cells from activated $CD4^+CD25^-$ Teff cells treated with either vehicle or UK5099. LeanNGT (n = 11, green dots), ObNGT (n = 9, blue dots), and T2D (n = 9, red dots). Differences were assessed by a one-way ANOVA (A-C) or two-way ANOVA (D-F) and Bonferroni's multiple comparisons. *: $p < 0.05$, **: $p < 0.01$, ***: $p < 0.001$, ****: $p < 0.0001$
